# Prediction of functional microexons by transfer learning

**DOI:** 10.1186/s12864-021-08187-9

**Published:** 2021-11-26

**Authors:** Qi Cheng, Bo He, Chengkui Zhao, Hongyuan Bi, Duojiao Chen, Shuangze Han, Haikuan Gao, Weixing Feng

**Affiliations:** College of Intelligent Systems Science and Engineering, Harbin Engineering University, Harbin, China

**Keywords:** Microexon, Microindel, Transfer learning, Functional prediction

## Abstract

**Background:**

Microexons are a particular kind of exon of less than 30 nucleotides in length. More than 60% of annotated human microexons were found to have high levels of sequence conservation, suggesting their potential functions. There is thus a need to develop a method for predicting functional microexons.

**Results:**

Given the lack of a publicly available functional label for microexons, we employed a transfer learning skill called Transfer Component Analysis (TCA) to transfer the knowledge obtained from feature mapping for the prediction of functional microexons. To provide reference knowledge, microindels were chosen because of their similarities to microexons. Then, Support Vector Machine (SVM) was used to train a classification model in the newly built feature space for the functional microindels. With the trained model, functional microexons were predicted. We also built a tool based on this model to predict other functional microexons. We then used this tool to predict a total of 19 functional microexons reported in the literature. This approach successfully predicted 16 out of 19 samples, giving accuracy greater than 80%.

**Conclusions:**

In this study, we proposed a method for predicting functional microexons and applied it, with the predictive results being largely consistent with records in the literature.

## Background

With the development of RNA sequencing and corresponding computational tools, a specific kind of exon called microexon (3–30 nucleotides (nt) in length) was found, which has been attracting increasing interests [1–3]. In 2014, Irimia et al. designed VAST-TOOLS to analyze vertebrate alternative splicing (AS) and identified 696 AS microexons (3–27 nt) in 603 genes [4]. Li then developed ATMap and identified 13,145 constitutive and AS microexons of 6–51 nt [5]. In the same year, Yan reported 2,008 AS microexons (6–30 nt) from 1,587 genes [6]. These studies revealed that microexons are more prevalent and present in many more genes than previously anticipated. Given this abundance of microexons, there is a need to develop a method to predict potentially functional microexons.

More than 60% of annotated human microexons exhibit high levels of sequence conservation, suggestive of potential functions [2]. Compared with normal exons, microexons with the short length of less than 30 nt more frequently result in exon skipping, which confers a clear transcriptional disadvantage [7, 8]. This is consistent with the observation that AS exons are generally much shorter than constitutive ones. However, amino acid sequences affected by synonymous AS microexons show striking enrichment in protein domains involved in protein–protein interactions, which are parts of stable protein complexes and frequently act as central nodes in protein interaction networks [4, 5]. Several studies have indicated that the inclusion of microexons leads to changes in unstructured and disordered regions of proteins and remodels protein interaction networks. Meanwhile, AS microexons also affect protein functions in a tissue-specific manner. Despite their small size, microexons were found to play crucial roles in transcriptional and translational regulation through alternative splicing [3].

However, the insufficient data on functional labels of microexons make the task of predicting functional microexons difficult. This represents a typical machine learning problem because the acquisition of labeled data is often difficult. To solve this problem, recently, transfer learning has been developed through transferring sharable knowledge across different but related kinds of data to make the learning task feasible [9]. Here, we used a transfer learning method to design a model for identifying functional microexons. Taking account of the many similarities between microindels and microexons, we chose microindels as the source and employed a transfer learning skill called Transfer Component Analysis (TCA) to transfer the knowledge upon feature mapping for the prediction of functional microexons. First, we analyzed the characteristics of microexons and microindels from two perspectives: the transcriptional and translational levels. Then, we mapped the retrieved features from both microexons and microindels into a new feature space simultaneously with TCA. This process minimized the difference between the distributions of the two data sets while preserving the main properties of the data in the newly built space. After that, Support Vector Machine (SVM) was adopted to train the model with the transferred features of microindels as input. Finally, the trained model could predict functional microexons. In this approach, for a new microexon, the distance to each of our microexons would be computed using K-Nearest Neighbor (KNN) and its label would be predicted according K nearest labeled microexons in our data. To test this method, we collected 19 functional microexons reported in various papers [10–15]. According to our predictive results, 16 microexons were successfully recognized. This shows the feasibility of the predictive method based on TCA.

## Data and Methods

### Selection of source domain

**Fig. 1:**
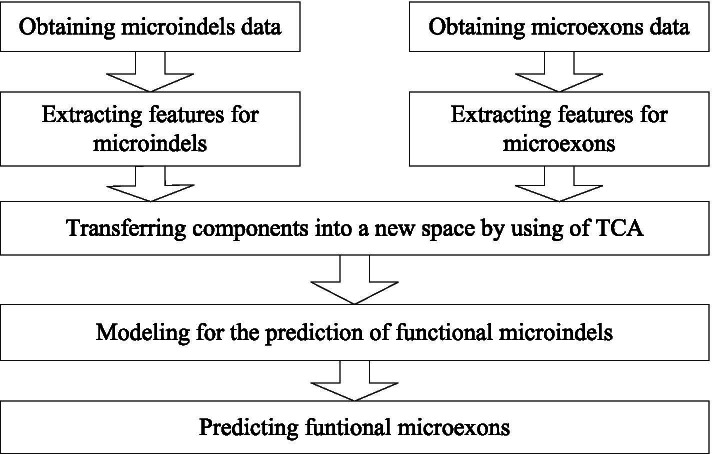
The process of functional microexons prediction by transfer learning

Because knowledge from a source domain is the basis of classification of the target domain, it is important to select a suitable source domain for transfer learning. The presence of more factors in common between the two different domains makes it easier to perform the transfer learning. In this study, we selected microindels as the source domain, from which knowledge is transferred to the prediction of microexons. There are four reasons for this choice. (1) Both microindels and microexons are small segments in genes, which have similar sizes and components. (2) Microindels and microexons have similar effects on transcription. Exons smaller than 50 nt can more easily undergo AS events than larger ones, so they are often included or skipped in gene sequences. This constitutes a resemblance to microindels. (3) Evidence shows that functional microindels and functional microexons have similar characteristics, such as being highly evolutionarily conserved, having a low probability of disorder, and exhibiting switch-like regulation. (4) Zhou has already proposed a model for predicting functional microindels called DDIG-in, which is known to exhibit good performance [16]. It is thus a good basis for predicting microexons. The process of predicting functional microexons by transfer learning is shown in Fig. 1.

### Data

In this study, two kinds of data needed to be collected. One was about microindels, and the other was about microexons. For data on microindels, the positive (pathogenic or functional) data came from the HGMD [17]^1^, and we obtained 2,036 non-frameshifting microindels(NFS-microindels) involving an insertion/deletion shorter than 30 nucleotides in multiples of three nucleotides. Of those, 1,694 and 342 were microdeletions and microinsertions, respectively. The negative data were from the 1000 Genomes Project [18]. Similarly, we obtained a total of 2,546 neutral microindels, including 1,806 microdeletions and 740 microinsertions.

The data about microexons were retrieved from hg19 in the Ensemble database [19]. It was reported that exons smaller than 30 nt have a clear transcriptional disadvantage according to the molecular dynamics of the splicing machinery, which frequently results in exon skipping. After excluding the frameshift microexons and those located in introns or containing stop codons, we obtained 3,941 microexons, among which AS was found in 3,714 microexons, namely, 94.2% of the total.

### Feature extraction

We extracted features for microindels and microexons from two perspectives: the gene level and the protein level. All extracted features are listed in Table 1. We selected two kinds of feature from the gene sequences, exon length and DNA conservation score, where the DNA conservation scores were from phylop (phylogenetic p-values) in UCSC [20, 21]^2^. To obtain DNA conservation scores, we calculated maximum, minimum, and average DNA conservation scores containing complete microexons/microindels area plus a fix before and after windows with n_window_=2. Namely, the numbers of bases used for the calculation of DNA conservation scores were as follows: n_del_+2n_window_ for microdeletions, 2+2n_window_ for microinsertions, and n_exon_+2n_window_ for microexons. At the protein level, structural characteristics of proteins coded by microindels and microexons were predicted by a series of SPINE tools, where secondary structures included α-helix (H), β-sheet (E), and random coil (C), and accessible surface areas (ASA) were predicted by SPINE-X and disorder scores were predicted by SPINE-D [22–24]. Similar to the DNA conservation scores, we also considered the target area before and after 2n_window_ residues. In addition, we examined three length-related features, namely, protein length, and distances to the protein amino and carboxyl terminal ends.

**Table 1:** The extracted features

Features	Description
***Gene level***	
Length (1)	Microindels/microexons length
DNA conservation scores (3)	Maximum, minimum, average
***Protein level***	
Secondary structure probabilities (12)	Maximum, minimum, average probability (C, H, E), Predicted secondary structure (C, H, E)
Disorder scores (3)	Maximum, minimum, average
ASA(3)	Maximum, minimum, average
Lengths (3)	Protein length, Distances to terminals (start and end)

Overall, we selected six different features at the gene and protein levels and extracted 25 different features

### Component transfer

For transfer learning, the prediction can be thought of as involving the learning of knowledge from training data and using that knowledge to classify the target data. That is, the source domain can be considered as the training set and the target domain as the testing set. To reduce differences between source and target domain, it is necessary to build a new feature space where the mapping features from both source and target domain data have identical distributions. So, our task is to look for a mapping method to build a new space to reduce the difference between the distributions of source and target mapping features while preserving the main properties of source and target data.

We used the transfer learning skill called Transfer Component Analysis (TCA) to accomplish this task. To learn transfer components underlying both source and target domain features to build a new feature space, the distance between the two feature distributions of microindels and microexons was measured using the empirical means of the two distributions as follows:

1 $$Dist({X_S},{X_T})=\left\| {\frac{1}{{{n_1}}}\sum\limits_{{i=1}}^{{{n_1}}} {\phi ({x_{{S_i}}})} - \frac{1}{{{n_2}}}\sum\limits_{{i=1}}^{{{n_2}}} {\phi ({x_{{T_i}}})} } \right\|_{{\rm H}}^{2}$$

where *X*_*S*_ and *X*_*T*_ are the microindels’ and microexons’ original features; ${\left\| \cdot \right\|_{\rm H}}$ is the form of a reproducing kernel in Hilbert space; and $\phi$ is a nonlinear mapping function, which embeds both the resource and target domain data into a shared low-dimensional latent space.

Specifically, let the Gram matrices defined on the source domain, target domain, and cross-domain in the embedded space be *K*_*S,S*_, *K*_*S,T*_, *K*_*T,S*_, and *K*_*T,T*_. So, they can be concatenated as matrix *K*.

2 $$K=\left[ {\begin{array}{*{20}{c}} {{K_{S,S}}}&{{K_{S,T}}} \\ {{K_{T,S}}}&{{K_{T,T}}} \end{array}} \right] \in {{\mathbb{R}}^{({n_1}+{n_2}) \times ({n_1}+{n_2})}}$$

For this, the objective function is set to minimize the distance between the projected source and target domain data while maximizing the variance of the embedded data as follows:

3 $$\begin{array}{*{20}{c}} {\mathop {max}\limits_{{K \geqslant 0}} }&{tr(KL) - \lambda tr(K)} \end{array}$$

where $${L_{ij}}=\left\{ {\begin{array}{*{20}{c}} {{1 \mathord{\left/ {\vphantom {1 {n_{1}^{2}}}} \right. \kern-0pt} {n_{1}^{2}}},{x_i},{x_j} \in {X_{src}}} \\ {{1 \mathord{\left/ {\vphantom {1 {n_{2}^{2}}}} \right. \kern-0pt} {n_{2}^{2}}},{x_i},{x_j} \in {X_{tar}}} \\ {{{ - 1} \mathord{\left/ {\vphantom {{ - 1} {\left( {{n_1}{n_2}} \right)}}} \right. \kern-0pt} {\left( {{n_1}{n_2}} \right)}},{\text{otherwise}}} \end{array}} \right.$$and$\lambda \geqslant 0$ is a tradeoff parameter.

Then, to reduce computational complexity, the problem is simplified to compute the first *m* nonzero eigenvectors of the following matrix:

4 $${\left( {KLK+\mu I} \right)^{ - 1}}KHK$$

where $H={I_{{n_1}+{n_2}}} - {1 \mathord{\left/ {\vphantom {1 {\left( {{n_1}+{n_2}} \right)}}} \right. \kern-0pt} {\left( {{n_1}+{n_2}} \right)}}{\mathbf{1}}{{\mathbf{1}}^{\text{T}}}$ is the centering matrix, *I* is the identity matrix,${\mathbf{1}} \in {{\mathbb{R}}^{{n_1}+{n_2}}}$ is the column vector with all 1, *µ* is the nonzero coefficient to ensure that Eq. (4) is viable mathematically, and *µ* = 0.1 in this project.

### Functional microexons prediction

In the newly built feature space, we used SVM to train the model upon the transferred components from microindels for the prediction of functional microexons. To prove the feasibility of transfer learning, 10-fold cross-validation was performed for the modeling based on SVM to evaluate the model. Then, in predicting functional microexons, the SVM was trained by all microindels in new latent space after TCA.

According to the description of TCA above, it is a kind of feature mapping with a statistical method, which can only be used for feature mapping between data sets containing a large amount of data. It is difficult to find a mapping function that applies to a single sample to fit TCA. Therefore, for a new microexon, TCA cannot be used directly. We found almost all NFS-microexons with a length shorter than 30 nt and multiples of three nucleotides in HG19 and predicted their functional probability using TCA+SVM. However, some microexons remained undiscovered. Therefore, in our software, a new microexon’s label can be predicted by employing KNN with *k* = 5. First, the same method as Sect. 2.3 can be used to extracted features in Table 1 for the new microexon. Then, KNN is used to calculate the distance of the new microexon to each of our microexons, as labeled by TCA+SVM. Finally, the label of this new microexon is decided by the mean of the results of the five nearest microexons. We packaged this model as a publicly available tool that can be obtained at https://github.com/Cheng-qi/MicroexonPredict.

## Results and Discussion

### Distribution of data from source and target domains

For TCA, it is important to ensure the similarity of the distribution between microindels and microexons in the new latent space. Only when their distributions are sufficiently similar can microexons be considered as the testing set for microindels. Therefore, we first measured their distribution by using empirical means. The results indicated that the distance of their distribution is 0.092 in the new transferred latent space. Compared with 0.54 between the two original data spaces, this constitutes a reduction of about 83%. It is a great advantage of TCA that it can significantly reduce the difference between the source and target domains and then extract effective features on the basis of preserving characteristics of the data.

**Fig. 2:**
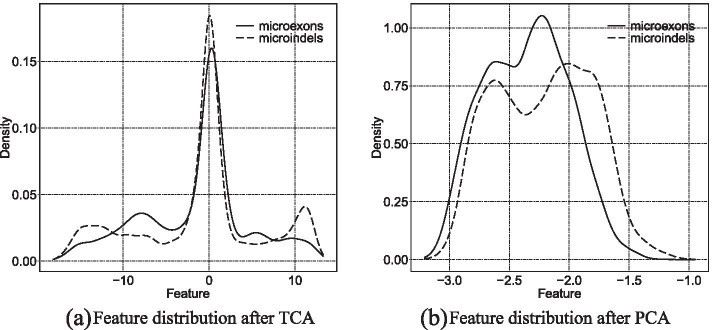
Data distribution of microexons and microindels in the transferred latent space based on TCA and PCA. (**a**) Feature distribution after TCA (**b**) Feature distribution after PCA

To prove the benefit of TCA, we also used Principal Components Analysis (PCA) to extract features. PCA is also a classical method to retrieve useful features from original data [25]. The feature distributions of microindels and microexons based on TCA and PCA are presented in Fig. 2. Compared with the result based on TCA, the distance based on PCA is clearly larger. This indicates that TCA is more effective at reducing the difference between two different domains.

### Prediction of microindels

**Fig. 3:**
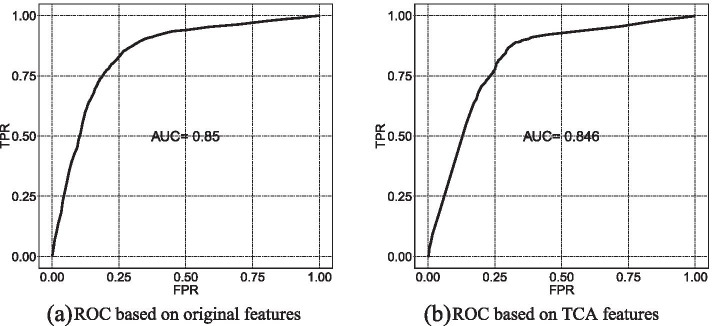
Comparison of ROC between the predictive results based on the original features (**a**) and TCA features (**b**)

To ensure that microexons can be predicted accurately, it is important that microindels can be predicted accurately after transferring. Therefore, we employed SVM to build two predictive models, one based on original features of the microindels and the other based on transferred components of the microindels. All predicted results are summarized in Table 2; Fig. 3.

**Table 2:** Performance for predicting microindels

	Precision	Acc	MCC	Recall	AUC
Original features	0.749	0.785	0.567	0.776	0.850
After TCA	0.713	0.769	0.542	0.804	0.846

In Table 2, the results of predicting microindels before and after TCA were evaluated using a 10-fold cross-validation method. First, we used the original features for modeling, obtaining precision of 74.9%, accuracy of 78.5%, MCC of 0.567, recall rate of 77.6%, and AUC of 0.85. Then, the features after TCA were trained in a new model, which achieved precision of 71.3%, accuracy of 76.9%, MCC of 0.542, recall rate of 80.4%, and AUC of 0.846. These results showed that, after TCA, regarding some comprehensive performance factors, ACC and MCC were only reduced by 1.6% and 0.25, respectively, and AUC was only reduced by 0.004. At the same time, the recall rate of the model increased by 2.8% after TCA. Overall, the model based on transferred components maintains good predictive activity, which benefited from the substantial preservation of data properties after TCA.

### Prediction of microexons

We mapped all the microindels and microexons to the new feature space using TCA. in new latent space after TCA, 3,941 microexons were classified using the SVM model trained by microindels. Of these, 2,021 microexons were labeled as functional, accounting for 51.3% of the total. This suggested that despite microexons being shorter than 30 nucleotides, they may play important roles in biological activities. Next, PCA was employed to analyze the contribution of each feature to the prediction of functional microexons, as shown in 4.

**Fig. 4:**
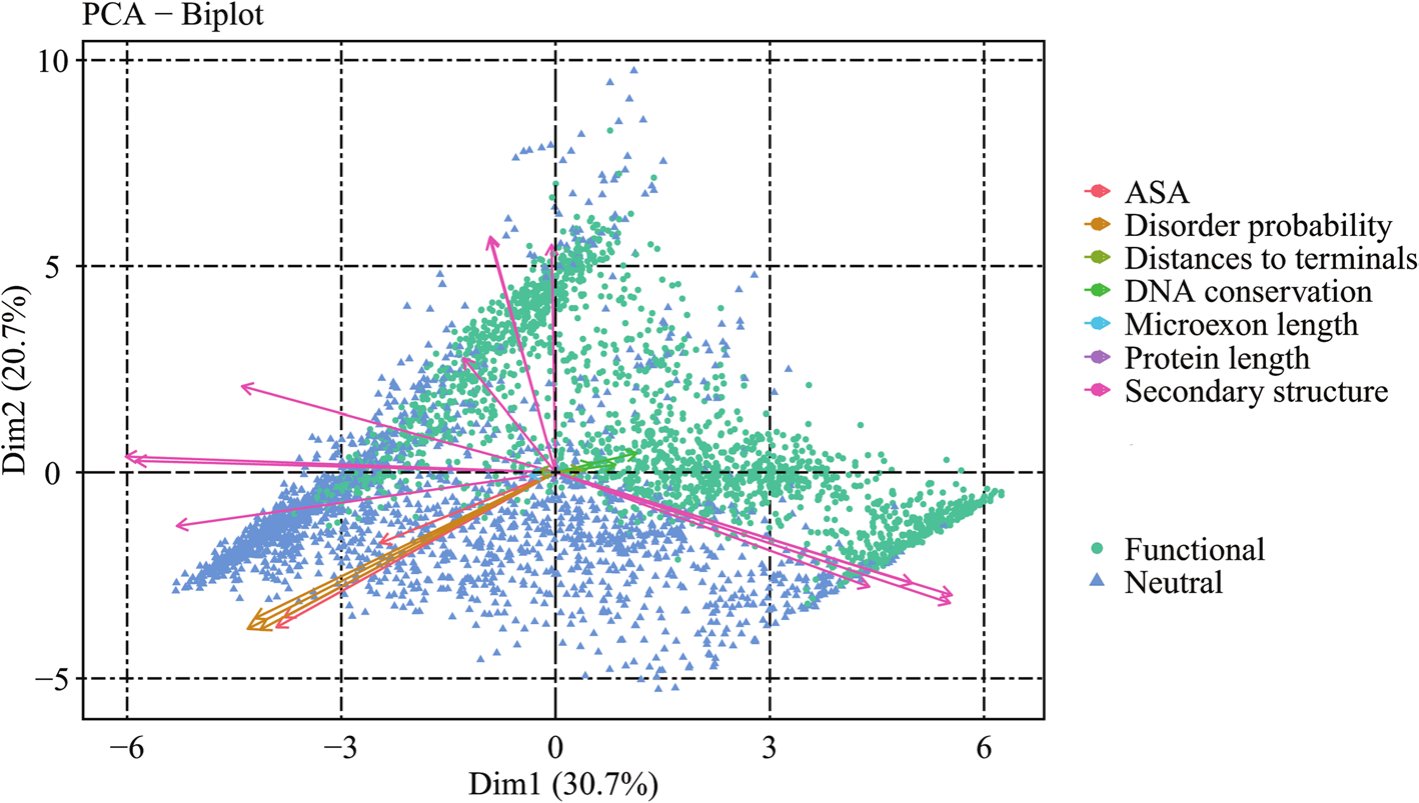
Contribution of different features in the PCA space to the prediction results

Figure 4 shows that disorder score, secondary structure probability, and ASA greatly influenced the prediction results of functional microexons, while DNA conservation and length had little influence on them. Focusing on these former three features, we conducted detailed analyses.

**Fig. 5:**
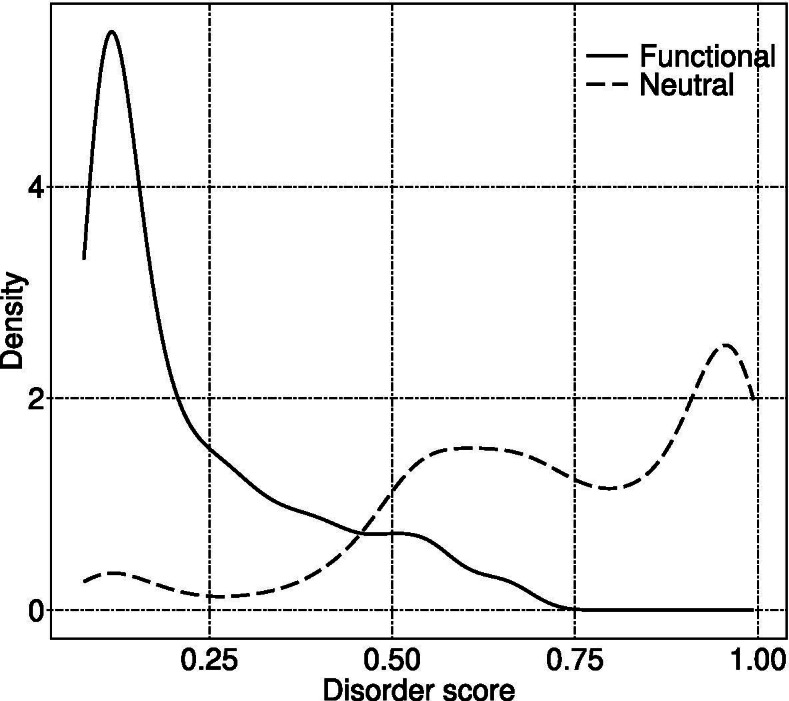
Distribution of average disorder scores of amino acid sequences encoded by microexons. For any microexon, the average disorder score is the mean value of the disorder scores for all amino acids encoded by it

Figure 5 depicts the density distribution of average disorder scores of amino acid sequences encoding microexons with different labels. It supports the view that most of the amino acid sequences encoded by functional microexons have lower disorder scores.

**Fig. 6:**
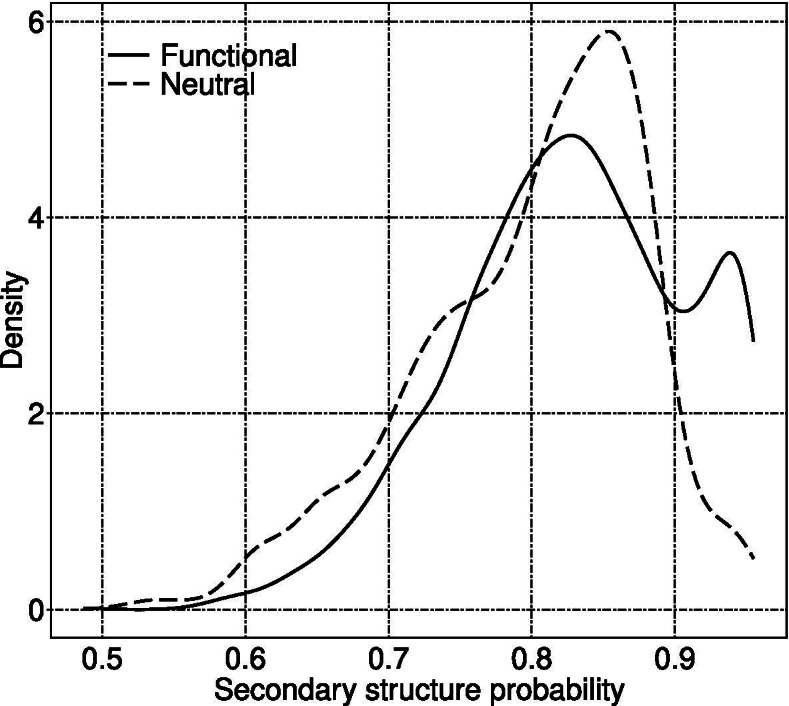
Distribution of average probability values of the most probable secondary structure (among C, H, and E) of amino acid sequences encoded by microexons. First, the maximum score of three secondary structures C, H, and E is chosen for each amino acid in an amino acid sequence. Then, the average value is calculated as the most probable secondary structure score. Finally, we obtain two distributions corresponding to functional and neutral microexons

We also analyzed the relationship between microexons with different labels and their secondary structure probability. As Fig. 6 shows, neutral microexons had two peaks of secondary structure probability at 0.83 and 0.97, while functional microexons had a single peak at 0.85, indicating that the microexons are most likely to be labeled as functional when their secondary structure probability is greater than 0.9. That is, microexons encoding proteins with a fixed secondary structure are more likely to be functional.

**Fig. 7:**
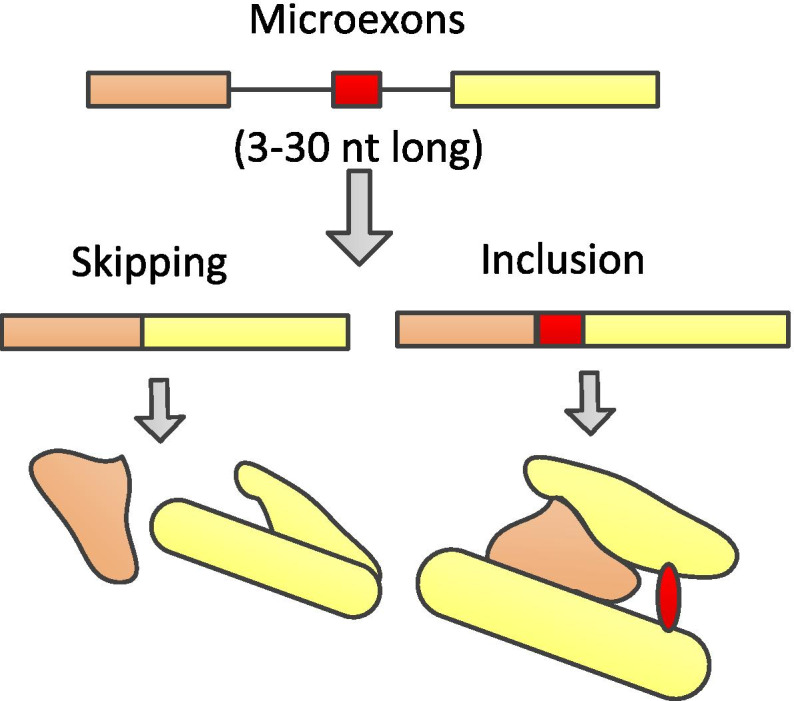
Diagram of the effects of microexons on protein domain structure. Inclusion of microexons leads to changes in protein structure

In fact, in some cases, amino acid sequences encoded by microexons can change the protein structure and show striking enrichment in protein domains, as shown in Fig. 7 [3]. Therefore, it can be concluded that functional microexons tend to be associated with stable protein structures.

**Fig. 8:**
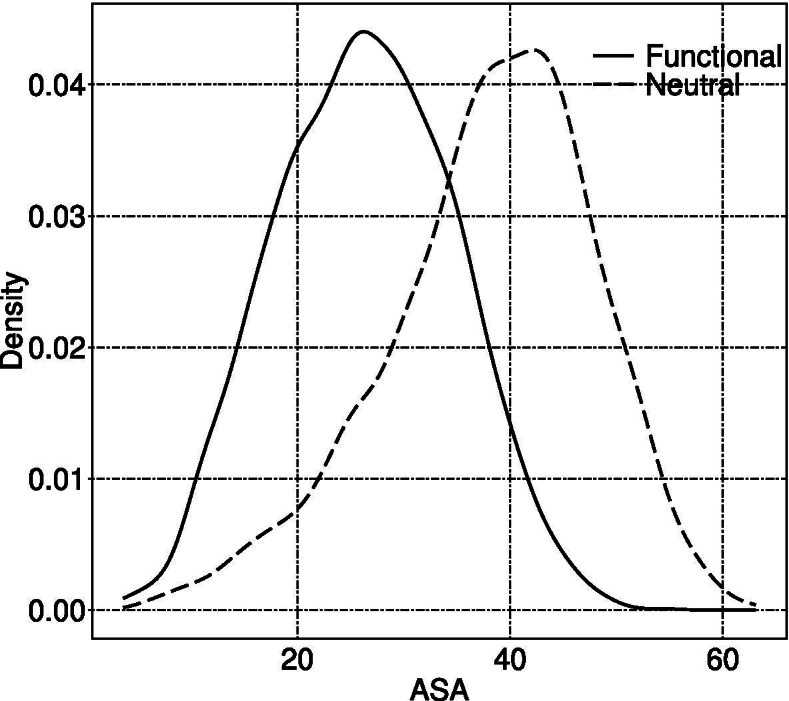
Distribution of average ASA values under different labels. We obtained these by calculating ASA mean values of amino acid sequences corresponding to microexons

As indicated in Fig. 4, ASA is also an important feature related to the prediction of functional microexons. So, as shown in Fig. 8, we analyzed the density distributions of ASA under different labels, which are approximately Gaussians. The peak of the ASA distribution of microexons labeled as functional is 25, but that of microexons labeled as neutral is 45. Therefore, it can be concluded that microexons with low ASA values are more likely to be functional than those with high ASA values.

### Some cases

To check the ability of our method to predict functional microexons, we found 19 functional microexons cases in some published literatures, and used our method to predict their functional labels. The predictive results are listed in Table 3. If the threshold value was set to 0.5, 16 out of 19 samples could be correctly predicted. Even when setting the threshold to 0.6, the number of correctly predicted samples was 15. This shows the feasibility of our method in the absence of sample labels.

**Table 3:** Results of microexons functional prediction of 19 cases reported in the literature

Gene name	Start (Based 0)	Len.	Prob.
ROBO1 [2]	chr3:78,696,778	27	0.739
ANK2 [2]	chr4:114,158,754	24	0.744
CR1 [26]	chr1:207,795,317	24	0.402
PICALM [27]	chr11:85,689,112	24	0.844
FERMT2 [26]	chr14:53,327,731	21	0.745
ITSN1 [10]	chr21:35,174,733	15	0.631
ZFYVE27 [11]	chr10:99,512,613	21	0.116
L1CAM [12]	chrX:153,141,083	21	0.811
DTNA [2]	chr18:32,401,063	9	0.581
SHANK2 [2]	chr11:70,788,698	12	0.711
APBB1 [4]	chr11:6,423,206	6	0.776
APBB2 [4]	chr4:40,824,046	21	0.802
APBB3 [4]	chr5:139,941,428	6	0.717
TRAPPC9 [4]	chr8:141,436,713	27	0.865
RAB3GAP1 [4]	chr2:135,809,868	27	0.731
Bin1 [26]	chr2:127,810,997	24	0.740
DOCK9 [4]	chr13:99,461,376	6	0.663
MEF2D [4]	chr1:156,446,285	21	0.120
KDM1A [28]	chr1:23,385,839	12	0.717

## Conclusions

To predict functional microexons, we employed transfer learning to create a low-dimensional latent space where the feature distributions between the obtained microexons and microindels were sufficiently close. In this new space, SVM was used to train a classification model for the functional microindels. With this trained model, functional microexons were predicted, with the prediction results being found to be consistent with records in literatures.

## Data Availability

All of open accessible data sets and software freely available at https://github.com/Cheng-qi/MicroexonPredict. An open online service can be found in http://MicroExonsPredict.chengqi.site/onlineSevice.

## Footnotes

1 Obtained from opened data in https://sparks-lab.org/server/ddig/ (Zhao, et al., 2013).

2
http://hgdownload.cse.ucsc.edu/goldenPath/hg19/phyloP46way.
